# Objective assessment of transition hyperactivated motility in 10-μm chambers: a functional predictor of sperm recovery in assisted reproduction

**DOI:** 10.3389/fendo.2026.1870772

**Published:** 2026-09-21

**Authors:** Gabriela Ruth Mendeluk, Herberto Repetto, Julia Ariagno, Verónica Alonso, Patricia Chenlo, Susana Curi

**Affiliations:** Laboratory of Male Fertility, Buenos Aires University Clinical Hospital “José de San Martín”, Instituto de Fisiopatología y Bioquímica Clínica (INFIBIOC), Faculty of Pharmacy and Biochemistry, University of Buenos Aires, Buenos Aires, Argentina

**Keywords:** 10-μm chamber, CASA, male infertility, sperm hyperactivation, thyroxine

## Abstract

**Background:**

Conventional sperm motility analysis often fails to capture the dynamic physiological changes associated with fertilization. The identification of a transition hyperactivated (TH) phenotype, characterized by high vigor while preserving linearity, may provide additional information on ejaculate functional competence beyond standard semen parameters.

**Objective:**

To establish an objective method for detecting TH motility using computer-assisted sperm analysis (CASA) with 10-μm chambers and to evaluate its clinical predictive value in comparison with pharmacological induction.

**Materials and methods:**

The Sperm Class Analyzer (SCA) system (Microptic SL) was validated using the following kinematic thresholds: curvilinear velocity (VCL) > 35 μm/s, linearity (LIN) > 50%, and amplitude of lateral head displacement (ALH) > 2.5 μm. Semen samples from fertile men (*n* = 27) and men consulting for infertility (*n* = 229) were analyzed under different conditions. TH motility was assessed in ejaculated semen (THE), after swim-up (THS), and after overnight incubation (THO). The non-genomic effects of thyroxine (T4, 0.002 μg/mL) on TH induction and on the number of total recovered spermatozoa (NTRS) were also evaluated.

**Results:**

Significant differences were observed among the analyzed conditions (Kruskal–Wallis test with Conover *post-hoc* analysis, *p* < 0.001). Fertile men showed no significant difference between THE and THO, whereas significant differences were observed in men consulting for infertility. THE was significantly higher in fertile men than in men with infertility [median (interquartile range, IQR): 8.2% (4.9–14.8) vs. 4.9% (2.7–9.0); *p* < 0.001]. THE values above the upper quartile of patients with infertility (> 9%) correlated with NTRS (Spearman’s *rs* = 0.779, *p* = 0.0004). In paired samples, T4 treatment significantly increased NTRS [median (IQR): 4.4 × 10^6^ (3.6–9.2) vs. 10.7 × 10^6^ (6.1–16.7); *p* = 0.00075], enabling samples to reach the threshold for intrauterine insemination.

**Conclusion:**

Transition hyperactivated motility represents a novel and objective functional biomarker of sperm kinematic competence beyond conventional semen parameters. Measurement of THE in 10-μm chambers provides a robust analytical approach for functional sperm assessment. Combined with T4 induction, this strategy may offer clinically relevant information on sperm functional status. Prospective studies including pregnancy outcomes are needed to determine its predictive value for clinical fertility and assisted reproduction.

## Introduction

Standard semen analysis, as codified in successive editions of the World Health Organization (WHO) manuals, includes computer-assisted sperm analysis (CASA) in Chapter 4, entitled “Advanced examination” (1), although CASA technology was introduced into andrology laboratories in the early 1980s (2). However, the routine clinical implementation of CASA remains limited by the lack of consensus guidelines for technical standardization and by the scarcity of external quality assessment programs that ensure interlaboratory comparability of diagnostic reports (3).

Hyperactivation, first characterized by Yanagimachi in 1969, is a specialized motility state defined by high-amplitude, asymmetrical flagellar beating (4). In humans, this phenomenon does not appear to be a uniform state but rather a dynamic process with distinct kinetic patterns (5). It may alternate between the classic “star-spin” pattern, often associated with the final approach to the oocyte, and a transition hyperactivation (TH) pattern (6). TH is characterized by rapid and vigorous movement that retains a certain degree of linearity, potentially allowing effective progression through cervical mucus and the uterotubal junction.

A major limitation in the objective study of TH is the technical environment in which the analysis is performed. Standard CASA protocols commonly use chambers with a depth of 20 μm. Although these chambers allow relatively unrestricted sperm movement, they may introduce tracking errors during high-amplitude oscillations because the sperm head frequently moves out of the focal plane. We hypothesize that chambers with a depth of 10 μm provide a more favorable hydrodynamic constraint that improves the capture of TH trajectories by CASA software, thereby increasing the fidelity of kinematic measurements (7).

The pharmacological modulation of this functional state is also of considerable clinical interest. Pentoxifylline, a phosphodiesterase inhibitor used to stimulate sperm motility, has shown variable effects across studies (8). In contrast, emerging evidence suggests that thyroxine (T4) may rapidly increase TH through non-genomic actions on the sperm membrane, possibly mediated by integrin receptors (αvβ3), with downstream effects on mitochondrial activity and calcium signaling (9, 10).

The present study aimed to establish TH as a functional biomarker of sperm competence by (1) determining TH values in men with proven fertility and in men consulting for infertility; (2) analyzing TH dynamics under capacitating conditions; and (3) evaluating the potential of T4 to improve sperm recovery rates for intrauterine insemination (IUI).

## Materials and methods

### Ethical approval and patient selection

This study was conducted in accordance with the Declaration of Helsinki and approved by the Ethics Committee of Hospital de Clínicas “José de San Martín” (Buenos Aires, Argentina). Written informed consent was obtained from all participants. The study included two groups:

- Group A (control): 27 semen samples from men with proven fertility, defined as having fathered a natural pregnancy resulting in a live birth within the 12 months before enrollment. Participants were aged 20–50 years.
- Group B (patients): 229 semen samples from men consulting for infertility.

Inclusion criteria: male partners of couples presenting with primary or secondary infertility (> 12 months of unprotected intercourse), aged 20–50 years. Exclusion criteria: severe azoospermia or extreme cryptozoospermia precluding reliable CASA motility tracking.

### Semen processing

Semen samples were obtained by masturbation after 2–7 days of sexual abstinence. After liquefaction (30 min at 37 °C), conventional semen parameters were assessed according to the WHO 2021 criteria. (1) Samples were then subjected to a swim-up procedure under sterile conditions. Briefly, after complete liquefaction, each semen sample was thoroughly homogenized and divided into two standard centrifuge tubes, with 0.5 mL of semen placed in each tube. The aliquots were gently overlaid with 1.5 mL of culture medium (Quinn’s Advantage Medium with HEPES; SAGE-*In Vitro* Fertilization, Inc., Trumbull, United States) while maintaining a distinct and undisturbed interface between the two phases. The tubes were incubated at 37 °C for 60 min in an inclined position of approximately 45° to allow motile spermatozoa to migrate into the upper phase. After incubation, the tubes were returned to a vertical position, and the upper phases were carefully aspirated and pooled into a single collection tube. The recovered fraction was centrifuged at 600 × g for 15 min, the supernatant was discarded, and the pellet was resuspended to a final volume of 0.5 mL with fresh medium before sperm count and kinematic evaluation.

To evaluate overnight transition hyperactivated motility (THO), sperm suspensions were incubated for exactly 24 h at 37°C in the same HEPES-buffered culture medium. Because the medium was HEPES-buffered, it maintained physiological pH during the 24-h experimental period without requiring a CO_2_-supplemented atmosphere.

### CASA kinematic configuration

Computer-assisted sperm analysis (CASA) was performed using Sperm Class Analyzer (SCA) software version 6.6.64 (Microptic S.L., Barcelona, Spain). A minimum of 400 spermatozoa were analyzed in each assay.

For motility and kinematic evaluation, acquisition parameters were set at a frame rate of 25 frames/s (25 Hz) with 25 images per track. The minimum number of track points was set at 10 to validate consecutive frame tracking. To exclude background drift and Brownian motion, the minimum curvilinear velocity (VCL) threshold was set at 5 μm/s.

The system was specifically standardized and validated for 10-μm Leja chambers (7). Based on our previous validation studies, the TH phenotype was defined using the following objective criteria (9):

- VCL (curvilinear velocity): > 35 μm/s
- LIN (linearity): > 50%
- ALH (amplitude of lateral head displacement): > 2.5 μm

### Pharmacological induction protocols

To evaluate the functional response to L-thyroxine (T4), 17 semen samples from men consulting for infertility were analyzed. Each sample was divided into two aliquots of 0.45 mL. A fresh hormone solution (0.02 μg/mL in phosphate-buffered saline [PBS], pH 7.2–7.4) was prepared from a stock solution of levothyroxine (Levotiroxina Montpellier, Bagó Argentine Group, Ciudad Autonoma de Buenos Aires, Argentine 200 μg/mL) under sterile conditions and protected from exposure to light and air to minimize degradation. Under aseptic conditions, 50 μL of the hormone solution was added to one aliquot (treated group), whereas 50 μL of vehicle buffer was added to the second aliquot (control group). The final T4 concentration was 0.002 μg/mL. This concentration was selected according to dose–response curves established in our patented protocol (11). To minimize observer bias, CASA video acquisition, kinematic tracking, and manual frame editing were performed by routine clinical laboratory personnel as part of standard diagnostic procedures.

### Statistical analysis

Data normality was assessed using the Shapiro–Wilk test. Continuous variables with a non-normal distribution are presented as median (interquartile range [IQR]: Q1–Q3). Group comparisons were performed using the Kruskal–Wallis test followed by Conover *post-hoc* analysis. Spearman’s rank correlation coefficient (*rs*) with 95% confidence intervals (CIs) was used to evaluate the association between semen parameters and the number of total recovered spermatozoa (NTRS) after swim-up, and the relationship between %THE and age. %THE > 9% (corresponding to the upper quartile, Q3, of the group with infertility) was used to identify patients with preserved kinematic potential, and the correlation between %THE and NTRS was assessed within this subgroup. The functional response to T4 was analyzed using the Wilcoxon signed-rank test. All analyses were performed using MedCalc^®^ Statistical Software version 20 (MedCalc Software Ltd, Ostend, Belgium). Statistical significance was defined as *p* < 0.05.

## Results

### Dynamics of transition hyperactivated motility in fertile men and in men consulting for infertility

To evaluate sperm functional behavior under capacitating conditions, we analyzed TH motility at three stages: baseline ejaculate (%THE), immediately after swim-up selection (%THS), and after overnight incubation under sterile capacitating conditions (%THO).

A highly significant shift in kinematic performance was observed after swim-up selection in both study populations (p < 0.001). In patients consulting for infertility, the median baseline %THE was 4.9% (IQR: 2.7–9.0), increasing significantly to 17.5% (IQR: 13.0–27.5) in the recovered swim-up fraction (%THS). In the fertile group, baseline %THE was significantly higher than that in the infertility group [8.2% (IQR: 4.9–14.8) vs. 4.9% (IQR: 2.7–9.0); p < 0.001]. After swim-up enrichment, median %THS increased to 19.4% (IQR: 8.8–28.4). Despite the marked difference in baseline %THE between fertile men and infertile patients, no statistically significant difference was observed between groups in the post-swim-up %THS values.

Distinct patterns emerged during overnight incubation. In fertile men, %THO decreased and returned to values that did not differ significantly from baseline %THE (Conover *post-hoc* analysis). In contrast, infertile patients maintained elevated TH levels overnight; %THO did not decrease significantly compared with %THS and remained significantly higher than baseline %THE (**Figure 1**).

**Figure 1:**
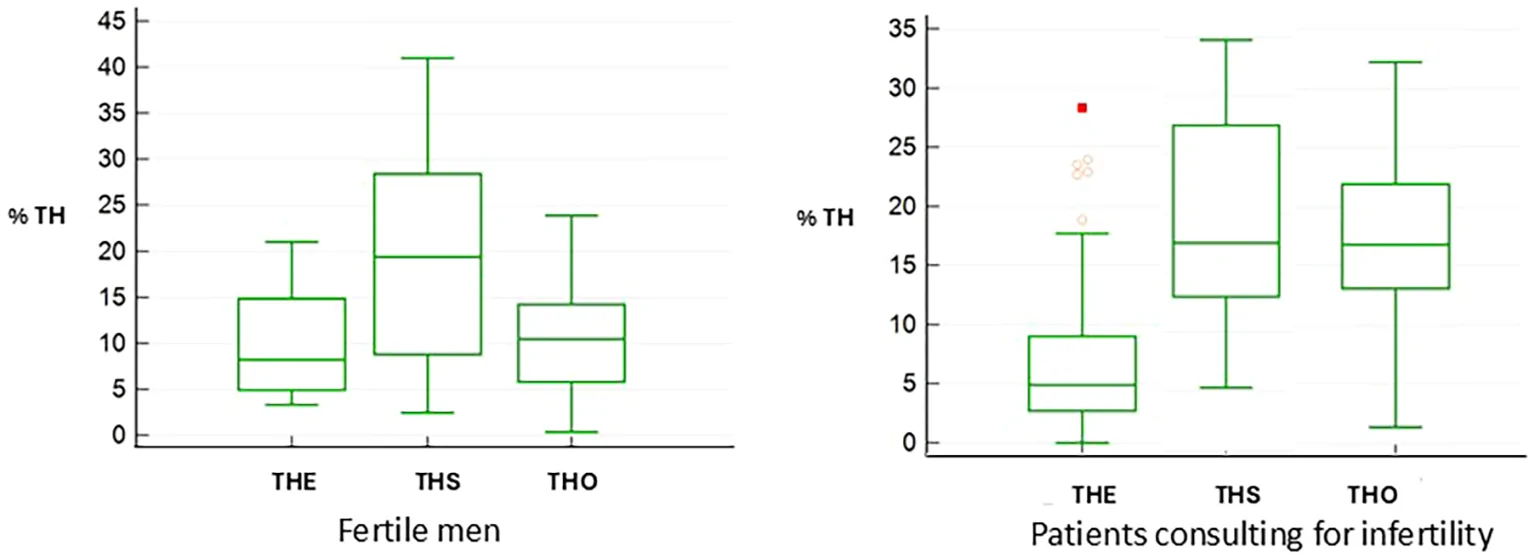
Distribution and dynamics of %TH across cohorts. Box-and-whisker plots showing baseline %THE, post-swim-up %THS, and overnight %THO in fertile controls (*n* = 27, left panel) and men consulting for infertility (*n* = 40, right panel). Boxes represent the 25th–75th percentiles, the horizontal line indicates the median, and whiskers represent the data range excluding outliers, which are shown individually. Statistical comparisons were performed using the Kruskal–Wallis test followed by Conover *post-hoc* analysis. Brackets indicate significant differences between conditions (*p* < 0.001).

### Longitudinal variations in fertile men and in patients consulting for infertility

Individual longitudinal analysis revealed distinct functional subphenotypes. In the fertile group, the majority of individuals showed a relatively homogeneous response, reaching post-swim-up %THS values clustered around a median of 20%, followed by a coordinated decrease after overnight incubation (**Figure 2**).

**Figure 2:**
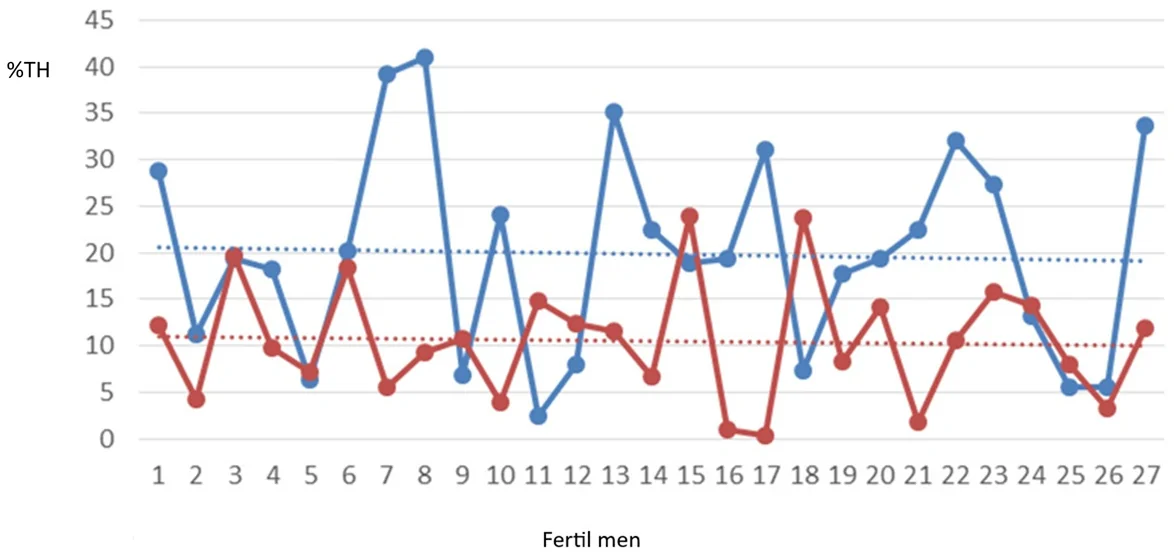
Temporal progression of %TH in fertile controls. Individual trajectories of %THS (blue circles) and %THO (red circles) in fertile controls (*n* = 27). Data points from the same individual are connected by solid lines to illustrate the progression from post-swim-up selection to overnight incubation. Dotted lines represent the overall linear regression trends for each condition, showing a consistent decrease in %TH after overnight incubation.

In contrast, the infertility group showed marked interindividual variability, with %THS values ranging from 4.2% to 39.2% (n = 24; **Figure 3**). Patients with a strong response to swim-up selection (%THS > 20%) subsequently exhibited a decrease in %THO, resembling the physiological pattern observed in fertile controls.

**Figure 3:**
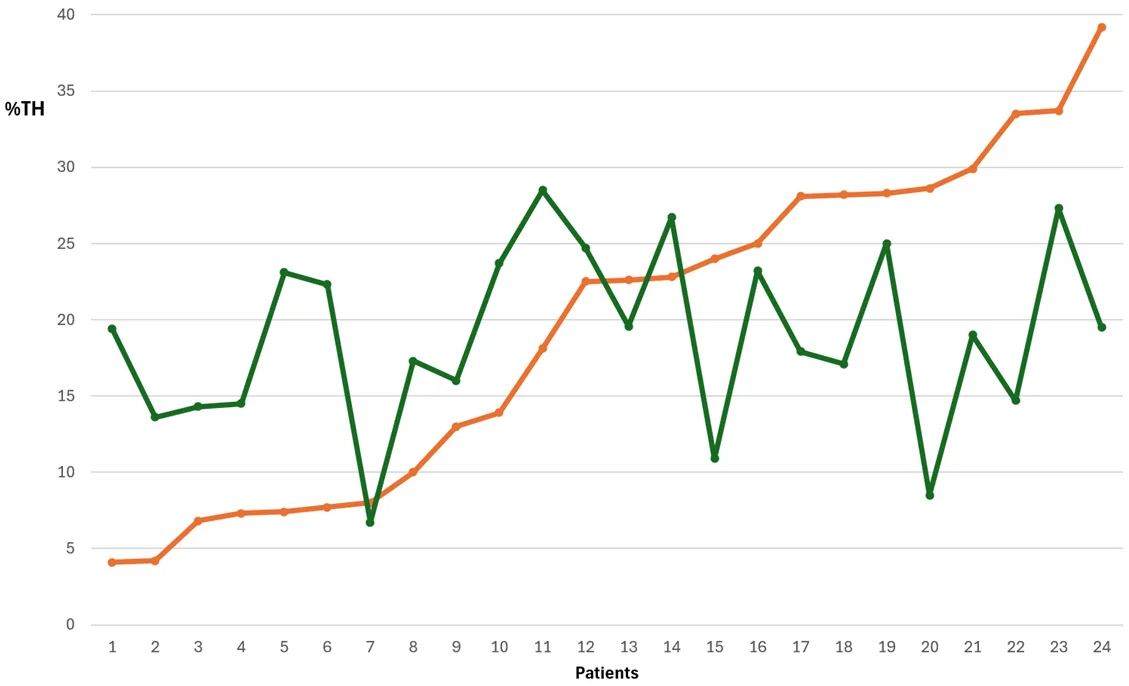
Temporal progression of %TH in patients consulting for infertility. Individual trajectories of %THS (orange circles) and %THO (green circles) in a subset of men consulting for infertility (*n* = 24). Individuals are ordered according to increasing %THS values to highlight distinct functional subphenotypes. Patients with higher post-swim-up values (> 20%) generally showed a decrease in %TH after overnight incubation, whereas Patient 7, who reported an abstinence period longer than 1 week, showed a flat trajectory below the functional threshold.

Two longitudinal patterns were identified among infertile patients: one resembling the fertile profile, with a decline in %THO after swim-up, and another characterized by persistent or even higher %THO values compared with %THS (**Figure 3**). Patient 7, who reported an abstinence period longer than 1 week, displayed a markedly flat kinematic profile, remaining below the selection threshold and showing no overnight activation.

### Paternal age and functional kinematics

To determine whether age influenced baseline ejaculate functional competence, we analyzed the relationship between paternal age and baseline %THE in 94 patients consulting for infertility. Spearman’s rank correlation analysis showed no significant association between age and %THE (rs = 0.0575, p = 0.5817), indicating that chronological age was not a reliable predictor of this functional biomarker (**Figure 4**).

**Figure 4:**
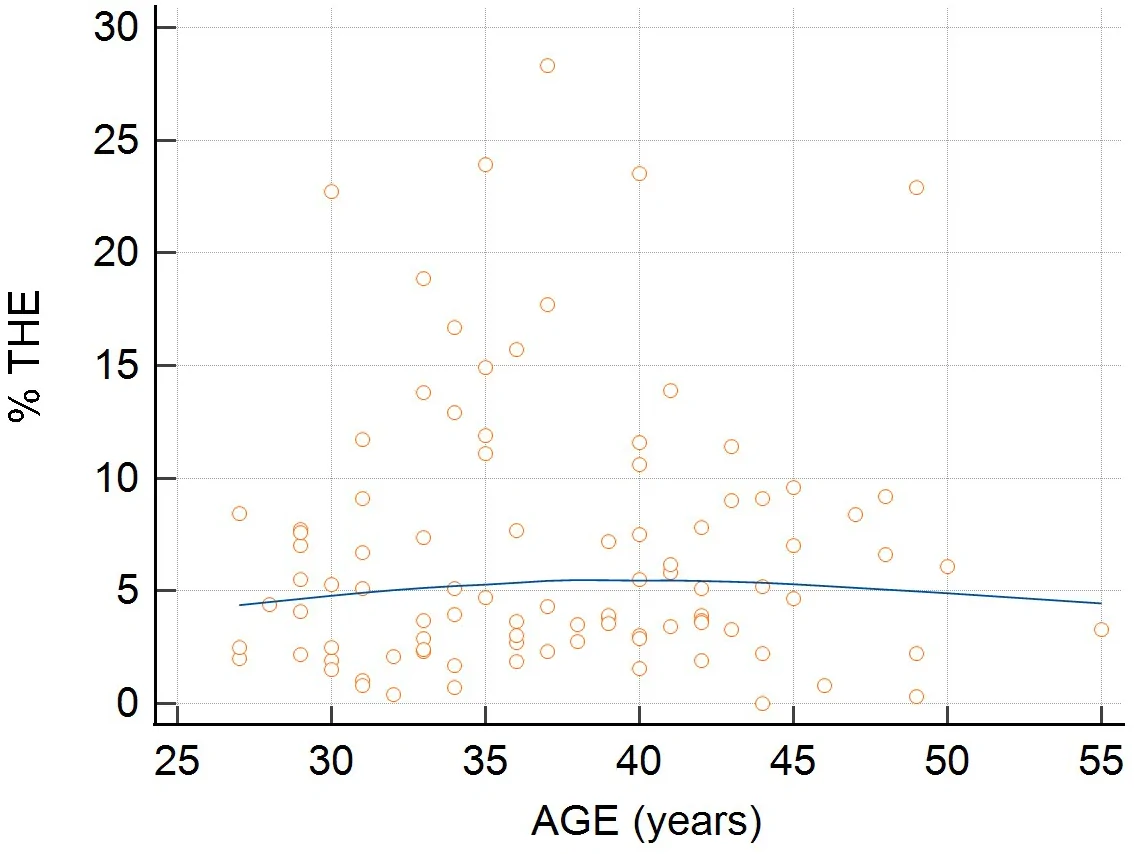
Baseline %THE in relation to paternal age. Scatter plot showing the relationship between baseline %THE and paternal age (years) in men consulting for infertility (*n* = 94). The blue line represents the linear regression trend. Spearman’s rank correlation analysis showed no significant association between age and %THE (*rs* = 0.0575; 95% CI: −0.147 to 0.257; *p* = 0.5817).

### Correlation of semen parameters and %THE with NTRS

To evaluate the potential clinical utility of these parameters for low-complexity assisted reproduction, we analyzed the correlations of conventional semen parameters and %THE with NTRS (*n* = 54; **Table 1**).

**Table 1:** Correlations between NTRS after swim-up and semen parameters.

Parameter	n	Spearman’s coefficient of rank correlation (rs) (95% CI)	p
Number of sperm/ejaculate	54	0.733 (0.579–0.837)	< 0.0001
Number of motile sperm/ejaculate	54	0.885 (0.810–0.932)	< 0.0001
Number of normal sperm/ejaculate	54	0.610 (0.409–0.754)	< 0.0001
THE/ejaculate	54	0.627 (0.431–0.766)	< 0.0001
%THE > 9%/ejaculate	16	0.779 (0.462–0.920)	0.0004

The *n* = 16 subgroup consisted of men consulting for infertility with %THE > 9% (the prespecified upper quartile [Q3] of the infertility cohort). In this subgroup, which showed preserved kinematic potential, baseline %THE was associated with higher post-wash sperm recovery.

NTRS showed a strong correlation with baseline sperm concentration and total motile sperm count, whereas moderate correlations were observed with sperm morphology and %THE. In addition, %THE values above the upper quartile threshold of 9% were associated with higher NTRS values.

This finding has direct clinical relevance: in our study population, 54.3% of patients (51/94) presented baseline %THE values below the 9% threshold (**Figure 4**), suggesting a reduced probability of achieving optimal post-swim-up sperm recovery for intrauterine insemination (IUI).

### Pharmacological rescue of borderline samples through non-genomic T4 induction

Finally, we evaluated the therapeutic potential of L-thyroxine (T4, 0.002 μg/mL) to pharmacologically improve samples with suboptimal recovery rates in infertile patients (*n* = 17). Exposure to the T4 protocol significantly increased NTRS from 4.4 × 10^6^ (IQR: 3.6–9.2) to 10.7 × 10^6^ (IQR: 6.1–16.7) (*p* = 0.00075). After T4 incubation, all evaluated samples reached the recommended threshold of 5 × 10^6^ total motile spermatozoa for IUI eligibility.

## Discussion

The transition from progressive motility to hyperactivation is likely one of the most relevant functional events in the sperm journey toward the oocyte. Our study demonstrates that TH can be objectively quantified using a standardized CASA configuration with 10-μm chambers. This technical choice is relevant because reducing chamber depth minimizes z-axis displacement and creates a more confined environment for kinematic tracking. This concept is consistent with emerging biophysical and microfluidic approaches that use controlled fluid dynamics to improvesperm selection in assisted reproduction (12).

The kinematic thresholds established for TH (VCL > 35 μm/s, LIN > 50%, and ALH > 2.5 μm) were selected to identify a specific functional subpopulation rather than to serve as arbitrary filtering criteria. A VCL above 35 μm/s together with an ALH above 2.5 μm suggests vigorous flagellar activity associated with increased energy demand. Importantly, the requirement of LIN > 50% distinguishes TH from the classical “star-spin” hyperactivation pattern. (5) From a functional perspective, this combination may identify spermatozoa capable of maintaining rapid forward progression while preserving the lateral head displacement required to move through the viscoelastic environment of the female reproductive tract (13–15).

This apparent kinetic equilibrium may reflect a state of high functional competence in which velocity and directional efficiency are simultaneously preserved. The observation that fertile men showed a median post-swim-up %THS close to 20% is in agreement with early observations reported by Burkman (16).

The main differences between fertile men and men consulting for infertility were observed at the ejaculate level. Fertile men showed significantly higher baseline %THE values, and these higher values were associated with higher NTRS. This association may have practical relevance when considering low-complexity assisted reproduction techniques. The marked increase in %THS observed in both groups most likely reflects the enrichment effect of the swim-up procedure itself (1).

An important conceptual question is why TH-positive spermatozoa are already present in the ejaculate, despite the accepted view that capacitation occurs mainly within the female genital tract or under specific *in vitro* conditions. We hypothesize that baseline %THE may reflect an intrinsic sperm property associated with fertilizing potential rather than complete capacitation.

A particularly interesting finding was that, in fertile men, %THO returned to values comparable to baseline %THE. One possible interpretation is that TH reflects the ability of spermatozoa to overcome the viscoelastic properties of female secretions, such as cervical mucus. Once this initial transit phase is completed, sustained high-linearity hyperactivation may no longer be required, suggesting a more efficient functional regulation in fertile samples. We further speculate that overnight incubation may favor a transition from TH to a more classical star-spin hyperactivation pattern that is not captured by our analytical configuration.

The longitudinal analysis also suggested heterogeneity among men consulting for infertility. As illustrated in **Figure 3**, two distinct TH dynamics were observed, one of which resembled the pattern identified in fertile men. This observation supports the concept that men consulting for infertility do not necessarily constitute a homogeneous population in terms of sperm functional competence.

Another relevant finding was the rapid effect of T4 on TH and sperm recovery. The significant increase observed after 20 min of exposure is compatible with a non-genomic mechanism of action (9). We hypothesize that T4 may enhance sperm functional activity through modulation of calcium signaling and/or mitochondrial ATP production. From a clinical perspective, this effect may be particularly relevant for samples with borderline recovery capacity, potentially improving eligibility for IUI. In our cohort, 51 of 94 patients presented baseline %THE values below the 9% threshold and may represent candidates for future evaluation of this pharmacological approach (11).

Previous studies by Condorelli et al. have shown beneficial effects of thyroid hormones on sperm mitochondrial function, oxidative stress, and DNA integrity (17). Our study extends these observations by introducing two additional aspects. First, we provide higher kinematic specificity by identifying a defined TH subpopulation rather than broad motility categories. Second, we propose baseline %THE as a potential functional biomarker associated with post-swim-up recovery quality. However, prospective studies including reproductive outcomes are required before %THE can be considered a predictive clinical marker.

Finally, the relationship between abstinence and TH deserves further investigation. Although some trends were observed in the present study, adequately powered population-based studies are needed to confirm or refute these preliminary findings (18, 19).

## Concluding remarks

TH motility represents a novel and objective functional biomarker that reflects sperm kinematic competence beyond conventional semen parameters. Measurement of %THE in 10-μm chambers provides a robust analytical approach for the functional assessment of male fertility and, when combined with T4 induction, may offer clinically relevant information on sperm functional status. Prospective studies including pregnancy and live-birth outcomes are needed to determine the predictive value of this approach for clinical fertility. Nevertheless, the proposed standardized framework may contribute to improving diagnostic accuracy and optimizing sperm selection strategies in assisted reproduction.

### Clinical and socioeconomic implications: shifting from high to low complexity

The identification of an objective threshold for TH motility may have implications for the clinical management of couples undergoing infertility evaluation. In current practice, some patients are referred to high-complexity treatments, such as intracytoplasmic sperm injection (ICSI), because conventional semen analysis does not provide sufficient information about sperm functional competence. Our findings suggest that a subset of these patients may retain measurable functional capacity, as reflected by baseline %THE and its response to T4 during sperm preparation.

From a health-system perspective, if future prospective studies confirm that TH-based assessment can improve patient stratification for intrauterine insemination (IUI), this approach may contribute to reducing the use of unnecessary high-complexity procedures. Such a strategy might be associated with lower treatment costs and a reduction in the burden associated with more invasive assisted reproduction techniques. In addition, the incorporation of objective functional biomarkers may help optimize laboratory decision-making during sperm preparation.

However, these potential clinical and socioeconomic benefits remain hypothetical and should be confirmed in prospective studies that include reproductive outcomes, cost-effectiveness analyses, and patient-centered measures.

## Conclusions

This study proposes an objective approach for the functional assessment of sperm motility based on TH quantification using 10-μm chambers. Combined with T4 induction, this strategy may provide additional information beyond conventional semen parameters.

Our findings suggest that TH assessment may be useful for the following:

- Stratifying patients according to functional kinematic characteristics rather than solely numerical semen parameters.
- Identifying differences in baseline %THE between fertile men and men consulting for infertility.
- Evaluating the relationship between TH and post-swim-up sperm recovery.
- Exploring the potential role of T4 in improving sperm recovery in samples with borderline functional capacity.

Prospective studies incorporating reproductive outcomes, including pregnancy and live-birth rates, are required to determine the clinical predictive value of this approach and its role in assisted reproduction.

## Study limitations

Several limitations of this study should be acknowledged.

Institutional and demographic constraints. This study was conducted in a tertiary university reference laboratory that receives specialized referral samples from multiple clinical centers across the country. As a consequence, detailed demographic and systemic variables (e.g., body mass index and metabolic profiles) and longitudinal reproductive follow-up data, including clinical pregnancy and live-birth outcomes after IUI or assisted reproductive technology (ART), were not systematically available for the entire historical cohort.

Single-center retrospective design. Although the cohort reflects more than two decades of clinical laboratory activity, the retrospective and single-center nature of the study may limit the generalizability of the absolute kinematic thresholds to laboratories using different CASA platforms, chamber systems, or tracking algorithms.

Heterogeneity of sample sizes across subcohorts. Because of differences in sample volume requirements, assay evolution, and operational protocols over time, different subcohorts were analyzed for specific objectives: infertility cohort (n = 229), TH kinematic characterization (n = 40), longitudinal analysis (n = 24), correlations of conventional semen parameters and %THE with NTRS (n = 54), and the T4 induction study (n = 17). This heterogeneity should be considered when interpreting the strength and precision of the associations observed.

Exploratory nature of the T4 findings. The T4 induction protocol produced a rapid increase in TH and sperm recovery under *in vitro* conditions, consistent with a potential non-genomic effect. However, these findings should be considered exploratory. Prospective clinical studies are required to determine whether T4-induced kinematic enhancement translates into improvements in fertilization, embryo development, pregnancy rates, and live-birth outcomes.

## Data Availability

The raw data supporting the conclusions of this article will be made available by the authors, without undue reservation.
